# Effectiveness of a multifaceted implementation strategy compared to usual care on low back pain guideline adherence among general practitioners

**DOI:** 10.1186/s12913-018-3166-y

**Published:** 2018-05-11

**Authors:** Arnela Suman, Frederieke G. Schaafsma, Peter M. van de Ven, Pauline Slottje, Rachelle Buchbinder, Maurits W. van Tulder, Johannes R. Anema

**Affiliations:** 1 Department of Public and Occupational Health, Amsterdam Public Health research institute, VU University medical centre, Amsterdam, The Netherlands; 2 Department of Public and Occupational Health, Amsterdam Public Health research institute, VU University medical centre, Amsterdam, The Netherlands, and the Research Centre for Insurance Medicine, Collaboration between AMC-UMCG-UWV-VUmc, Amsterdam, The Netherlands; 3 Department of Epidemiology and Biostatistics, Amsterdam Public Health research institute, VU University medical centre, Amsterdam, The Netherlands; 4 Department of General Practice and Elderly Care Medicine, Academic Network of General Practice (ANH), Amsterdam Public Health research institute, VU University medical centre, Amsterdam, The Netherlands; 5 Monash Department of Clinical Epidemiology, Cabrini Institute and Department of Epidemiology and Preventive Medicine, School of Public Health and Preventive Medicine, Monash University, Melbourne, Australia; 6 Department of Health Sciences, Faculty of Science, Vrije Universiteit, Amsterdam Movement Science research institute, Amsterdam, The Netherlands

**Keywords:** Primary health care, Low back pain, Health plan implementation, Guidelines, Referral and consultation

## Abstract

**Background:**

To improve patient care, and to reduce unnecessary referrals for diagnostic imaging and medical specialist care for low back pain, an evidence-based guideline for low back pain was developed in the Netherlands in 2010. The current study evaluated the effect of a multifaceted implementation strategy on guideline adherence among Dutch general practitioners.

**Methods:**

The implementation strategy included a multidisciplinary training, provision of educational material and an interactive website for healthcare professionals, supported by a multimedia eHealth intervention for patients with low back pain. Adherence was measured using performance indicators based on 3 months data extracted from the contacts with patients with low back pain recorded in the electronic medical records of participating general practitioners. Performance indicators were compared between two groups: a usual care group and an implementation group. Performance indicators were referrals to consultations with medical specialists, to diagnostic imaging, and to psychosocial and/or occupational physician consultations, and inquiries about psychosocial and occupational risk factors.

**Results:**

The electronic medical records of 5130 patient contacts for LBP were analysed; 2453 patient contacts in the usual care group and 2677 patient contacts in the implementation group. Overall, rates of referral and of recorded inquiries regarding psychosocial and occupational risk factors remained low in both groups over time. The only statistically significant difference found was a reduction in the number of referrals to neurologists in the implementation group (from 100 (7%) to 50 (4%)) compared to the usual care group (from 48 (4%) to 50 (4%), (*p* < 0.01)). There were no other between-group differences in referrals.

**Conclusion:**

In the short term, the strategy did not result in improved guideline adherence among general practitioners, and it is not recommended for widespread use. However, baseline referral rates in participating practices were already low, possibly leaving only little room for improvement. Inquiries for psychosocial and occupational risk factors remained low and this leaves room for improvement.

**Trial registration:**

This trial is registered in the Netherlands Trial Register (NTR): NTR4329. Registration date: December 20th, 2013.

## Background

Low back pain (LBP) is the leading cause of disability worldwide [1]. In the Netherlands, LBP and neck pain were the most prevalent disorders in 2015, with a year prevalence of 2 million among a total population of 16.7 million [2]. In the same year, almost 587,000 new cases of LBP were reported, making it also the disorder with the highest incidence [3]. The majority of LBP cases cannot be attributed to a specific underlying pathophysiological cause and are thus referred to as non-specific [4, 5].

Non-specific LBP is one of the most common conditions for which people in high-income countries seek medical care [6]. In the Netherlands in 2011, over 90% of patients registered at a GP practice that are known to have LBP contacted their general practitioner (GP) at least once per year regarding their LBP. A number of patients also seek help from other professionals (e.g. physiotherapist), seek only help from other professionals than their GP, or do not seek healthcare at all. In 2008, about 32% of LBP patients who visited their GP were referred to a medical specialist [7, 8]. Sixty per cent of these referrals were to neurology, 29% to orthopaedics, and 11% to neurosurgery [8]. Internationally, almost half of LBP patients (42%) are referred for LBP diagnostic imaging within 1 year of their first visit to a physician [9]. Research suggests that consultations with medical specialists often lead to further medical procedures, including diagnostic imaging [9]. Furthermore, there is evidence indicating that diagnostic imaging for LBP without any suggestion of a serious underlying cause, i.e. non-specific LBP, does not improve patient outcomes, and can in fact be harmful to patients, due to for example the risks of radiation and labelling of patients [9, 10].

To improve patient care by focusing more on psychosocial and occupational risk factors for LBP, and to reduce unnecessary referrals for diagnostic imaging and medical specialist care for non-specific LBP, an evidence-based guideline for LBP was developed in the Netherlands in 2010 [11]. The current study describes the effects of a multifaceted strategy to implement this guideline in Dutch general practice. Specifically, guideline adherence of GPs is evaluated by means of performance indicators based on the guideline, and the effectiveness of the implementation strategy is compared to usual care in a before-after design. Indicators and outcomes include: referrals to medical specialist care, referrals for diagnostic imaging, inquiries about psychosocial and work-related risk factors, and referrals for psychosocial consultation and to occupational physicians.

## Methods

This controlled before-after study was part of a stepped-wedge cluster-randomised controlled trial (RCT), that was registered in 2013 with the Netherlands Trial Register (NTR) under number NTR4329. The Medical Ethics Committee of the VU University medical centre assessed this study design and procedures, and in accordance with the local regulatory guidelines and standards for human subjects protection in the Netherlands (Medical Research Involving Human Subjects Act [WMO], 2005), this study proved to be exempt from further medical ethical review.

### Study design

The current paper reports the effectiveness of the implementation strategy on guideline adherence of GPs. While the study initially had a stepped-wedge design, it was not feasible to perform the evaluation of the guideline adherence by GPs according to this design [12]. Therefore, these outcomes were evaluated in a controlled before-after design. For the present analysis, the general practices were randomly ordered into two groups: an implementation group and a usual care group. The implementation group was studied by comparing a baseline period of 3 months (January–March 2014) at the start of the study (prior to the start of the implementation) and a follow-up period covering the same calendar period 1 year later, i.e. the first 3 months after the general practices in this group had received the implementation strategy. The usual care group was studied by comparing a baseline period of 3 months during the year prior to the start of the study (October–December 2013) and a follow-period covering the same calendar period 1 year later (prior to the start of the implementation in this group). The periods were different for the two groups due to the randomisation and the planned start and timing of the intervention. Electronic medical records (EMRs) of patients with LBP who contacted or visited the participating GPs were reviewed, and performance indicators extracted from these records were compared between these two groups.

### Implementation strategy

The strategy for the active implementation of the guideline (implementation group) consisted of multicomponent, multidisciplinary continuing medical education (CME) training in which interdisciplinary communication and collaboration (i.e. between GPs, physiotherapists, and occupational physicians), and patient-physician communication were central themes as means to reduce referrals to medical specialist consultation and diagnostic imaging, and to increase consideration of psychosocial and occupational risk factors. Several additional components, for example online and offline educational materials, and social media platforms (i.e. forum, Twitter, Facebook) supplemented the CME training. This professional-based strategy was supported by a patient-based eHealth strategy consisting of informative video-messages, information on various topics regarding LBP (e.g. work, daily life), exercises, and social media platforms.

### Participants and study settings

GPs were eligible to participate in this study if they were practising within the municipality of Amsterdam, and if they had patients with LBP registered in their practice. GPs were recruited within the Academic Network of General Practice of the department of General Practice and Elderly Care Medicine at the VU University medical centre (ANH-VUmc). A detailed description of the recruitment procedure has been described elsewhere [13]. Anonymised information related to LBP contacts were extracted from the EMRs of all patients that contacted the participating GPs at least once regarding LBP (coded according to the International Classification for Primary Care (ICPC) as L02, L03, L86) in the selected 3 month periods, and who had a date of birth between the years 1939 and 1996 (i.e. aged 18–76 years).

### Outcomes

The outcomes for this study were measures of guideline adherence by GPs. Guideline adherence was assessed using performance indicators. These performance indicators were operationalisations of the recommendations made in the guideline. The indicators are presented in Table 1.

**Table 1:** Performance indicators based on guideline recommendations to measure guideline adherence among GPs [12]

Guideline recommendation	Performance indicator for LBP	Operationalization
A small proportion of patients will not recover with help from the primary care sector, these patients should be referred to secondary care	Referral to consultation with medical specialists (neurology, orthopaedics or other specialty)	Referrals as percentage of total consultations for LBP per GP, reported separately per specialty
Diagnostic imaging is not routinely indicated for acute non-specific LBP; Diagnostic imaging is not recommended for patients with chronic non-specific LBP	Referral for diagnostic imaging	Referrals for MRI, X-ray, CT, Dexa or ultrasound as percentage of total consultations for LBP per GP, reported separately for every imaging technique
Be alert to psychosocial risk factors that can influence the prognosis of LBP, and analyse these if recovery does not occur; Evaluation of psychosocial risk factors that can influence the prognosis of LBP is recommended	Inquiries about psychosocial risk factors	Consultations where psychosocial risk factors were discussed and reported, as percentage of total consultations for LBP per GP
Cognitive behavioural therapy is recommended for patients with cognitive (and) behavioural problems; Patients with LBP that do not recover within 2–3 weeks and have psychosocial risk factors should be referred to a psychologist	Referral for psychosocial care as indicator for multidisciplinary collaboration	Referrals as percentage of total consultations for LBP per GP
In employed patients with LBP a prognosis and recovery expectations for return to work should be discussed	Inquiries about work-related risk factors	Consultations where occupational risk factors were reported as percentage of total consultations for LBP per GP
The general practitioner and the occupational physician should contact each other to coordinate care if the patient’s recovery is stagnating	Referral to and/or contact with occupational physician as indicator for multidisciplinary collaboration*	Consultations where referral to and/or contact with occupational physician was made as percentage of total consultations for LBP per GP, reported separately for referral to and contact between GP and occupational physician

*In the Netherlands, all employers are obligated to ask the advice of an occupational physician in case of a sick-listed employee. The occupational physician has a consultation with an employee when he/she is sick-listed within 6 weeks of the first sick day. The occupational physician will advise both the employee and the employer on what steps need to be taken for a healthy return to work

Indicators were measured at the level of all GP-patient contacts (i.e. consultations, home visits, telephone consultations) regarding LBP per general practice in the given time period. To account for potential seasonal differences, the calendar time periods for the baseline and follow-up periods from which the LBP contacts were extracted from the EMRs were matched per group. The indicators were scored based on anonymised data extracted from the ANH-VUmc database. This database contains pseudonymised general practice care data from the EMRs of the general practices participating in ANH-VUmc, according to Dutch privacy legislation. From all patient records that fulfilled the selection criteria, the following data were selected for review: sex, age category (18–65 versus 65+ years), and the free text annotations made by the GP. The indicators were scored by reviewing the free text annotations from the selected records by two researchers (AS, FGS) blinded to the group and calendar period allocation. A first set of records was reviewed by AS and FGS to reach consensus over scoring of indicators. One researcher (AS) reviewed all other records.

### Statistical methods

Descriptive statistics were used to summarise and compare the demographic information of GPs in the implementation group and usual care group. Performance indicators were compared between groups using Generalized Estimating Equations (GEEs) with a logistic link function, a fixed effect for time (baseline/follow-up measurement period), group (implementation/usual care), and an interaction between time and group. An exchangeable correlation structure was used to take into account the correlation between outcomes within the same group. Unadjusted analyses assessing differences over time between implementation group and usual care group were performed first, followed by adjusted multivariable models to correct for potential confounders: average GP age per practice, average years of working as GP per practice, and proportion of patients aged 65+ per practice. Potential confounders were included in final adjusted models when at least one of the regression coefficients for the categorical variable time changed by more than 10% compared to the unadjusted analyses. *P*-values were considered statistically significant at < 0.05. All statistical procedures were performed in IBM SPSS Statistics 22.

## Results

### Effects of the strategy on GP guideline adherence

**Fig. 1:**
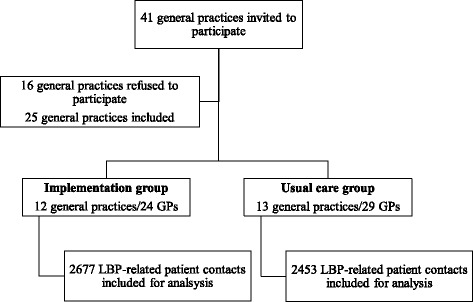
Flow-chart of inclusion process of GPs and EMRs

Twenty-five general practices, accounting for 53 individual GPs, participated in this study. The mean age of the participating GPs was 46.2 years (SD 10.2), 22 were male (41.5%), and they had an average of 14.3 (SD 9.3) years of work experience as GP. Within the time frame of data collection for this analysis, 19 GPs (from 11 practices) in the implementation group attended the CME training. All GPs, including those who did not attend the CME training, provided EMRs for analysis. These were 24 GPs from the implementation group, and 29 GPs from the usual care group. The EMRs of 5130 registered LBP contacts, belonging to 2549 unique LBP patients, were selected for review of performance indicators. Of these patients, 1515 were female (59.4%), and 328 (12.9%) patients were aged 65 years (statutory retirement age) or older. Figure 1 shows the flowchart of the inclusion process.

Table 2 shows the adjusted effects of the implementation strategy per performance indicator, i.e. the number of referrals and inquiries, respectively, as a proportion of total number of LBP patient contacts in each time period for both groups. In general, the numbers at baseline as well as the changes over time were small. The only statistically significant adjusted between-group difference over time was for the number of referrals to neurology. The number of referrals to neurologists decreased in the implementation group from 100 (7%) to 50 (4%), while it remained similar in the usual care group (48 referrals (4%) at baseline, and 50 referrals (4%) at follow-up), *p* < 0.01. There was no difference between groups over time with regards to the number of total referrals to medical specialist care. Total referral rates reduced over time from 171 (12%) to 100 (8%) in the implementation group, compared with 109 (9%) to 99 (8%) in the usual care group. The number of referrals to other medical specialities (e.g. rheumatology, rehabilitation, pain management) was very low and remained similar in both groups over time.

**Table 2:** Adjusted effects on performance indicators based on EMR of 5130 LBP patient contacts in all participating general practices

	Implementation group (*n* = 2677 LBP patient-GP contacts) [*n* = 1354 individual patients]		Usual care group (*n* = 2453 LBP patient-GP contacts) [*n* = 1195 individual patients)	
Indicator	Baseline (*n* = 1426 contacts) [*n* = 683 patients] Number (%)	Follow-up (*n* = 1251 contacts) [*n* = 671 patients] Number (%)	Baseline (*n* = 1211 contacts) [*n* = 580 patients] Number (%)	Follow-up (*n* = 1242 contacts) [*n* = 615 patients] Number (%)
Total referrals to specialists ^1^	171 (12)	100 (8)	109 (9)	99 (8)
Referrals to neurology ^1 ±^	100 (7)	50 (4)	48 (4)	50 (4)
Referrals to orthopaedics ^2^	0	0	12 (1)	0
Referrals to other specialities (e.g. Pain management, Rehabilitation medicine, Rheumatology)	29 (2)	38 (3)	36 (3)	37 (3)
Total imaging requests ^1^	200 (14)	138 (11)	145 (12)	137 (11)
MRI requests	43 (3)	25 (2)	24 (2)	25 (2)
X-ray requests ^3^	114 (8)	75 (6)	73 (6)	75 (6)
Other imaging requests (CT, Dexa, Ultrasound) *	4 (0.3)	9 (0.7)	6 (0.5)	4 (0.3)
Consideration of psychosocial risk factors	57 (4)	50 (4)	48 (4)	50 (4)
Referral for psychosocial care	5 (0.4)	5 (0.4)	4 (0.3)	4 (0.3)
Consideration of occupational risk factors	71 (5)	63 (5)	73 (6)	62 (5)
Referral to and/or contact with occupational physician	5 (0.4)	5 (0.4)	9 (0.7)	9 (0.7)

Numbers refer to 5130 patient-contacts belonging to 2549 unique LBP patients i.e. multiple contacts of the same patient over time were counted separately. 1: Adjusted for average years of experience in general practice; 2: Adjusted for number of patients aged 65+ years in GPs’ practices; 3: Adjusted for average age of GPs; * No time x group interaction estimate due to low number of events; ± *p* = 0.008

The overall rate of imaging requests decreased over time in both groups. While the decrease appeared larger in the implementation group (from 200 referrals (14%) at baseline to 138 referrals (11%) at follow up) than in the usual care group (from 145 referrals (12%) at baseline to 137 referrals (11%) at follow up), this difference was not statistically significant. There was also no statistically significant difference over time in the number of requests for specific imaging, although there appeared to be a decrease over time in the number of requests for X-rays in the implementation group (from 114 requests (8%) at baseline to 75 requests (6%)) while it remained the same in the usual care group over time (73 requests (6%) at baseline to 75 requests (6%) at follow-up). The number of requests for MRI imaging and other imaging techniques was very low in both groups at baseline, and remained similar over time.

There was no increase in either group over time in the number of times GPs registered consideration of psychosocial or occupational factors, or in the number of referrals for psychosocial care. The numbers of referrals to occupational physicians was very low in both groups at baseline and remained low over time.

## Discussion

This paper described a before-after study evaluating the effectiveness of a multifaceted strategy for the implementation of a multidisciplinary guideline for LBP in general practices in the Amsterdam area. The results of this study indicate that the strategy did not result in improvements in guideline adherence of GPs, as measured by performance indicators based on 5130 registered LBP patient contacts in the EMRs of GPs.

These results need to be interpreted within the context of the study population and GP behaviour changes that may have already occurred over time reducing the potential need and additional effect of implementing a LBP guideline in GP practice. On average, 32% of LBP patients visiting their GP in the Netherlands are referred to medical specialist care (numbers from 2008) [8]. In contrast, the GPs in the current study had an average referral rate to medical specialist care of only 9% per LBP contact at baseline in 2013. While the denominators of these percentages are not directly comparable (i.e. unique LBP patients versus LBP-GP contacts), it appears that our study population already had a lower referral rate compared to the nationwide average in 2008, which would mean there was less necessity to reduce their medical specialist referral rate [8]. Similarly, while nationwide 42% of patients that visited their GP for LBP were referred for diagnostic imaging in 2008, the average referral rate for diagnostic imaging in our study in 2013 through 2015 was only 12% per LBP-GP contact [8]. Referrals are not necessarily a bad clinical decision, but depend on the situation of the individual patient. Without extensive clinical data, it is not possible to rule out that the referral rates seen in the present study are inappropriate, but it is known that referrals for imaging and secondary care are not recommended for non-specific LBP [8, 11].

Conversely, at baseline occupational risk factors and psychosocial risk factors were only recorded in 5 and 4% of the LBP patient-GP contacts, respectively, indicating that improved guideline adherence regarding consideration of these risk factors for LBP is still a worthwhile goal. Research has shown that occupational and/or psychosocial factors can increase the risk of chronic LBP [14-16]. Paying more attention to these risk factors in individual patients to prevent chronicity, might improve patient outcomes, and reduce the prevalence of chronic LBP. It seems that since 2008, something has happened to improve GPs behaviour regarding referrals to medical specialists and diagnostic imaging, although this change apparently did not affect consideration of psychosocial or occupational aspects. It might be that the belief that referrals are unnecessary in many cases is now more widespread. It is unclear whether this is truly the case, or whether GPs are consistently not explicitly recording consideration of these aspects in EMRs, e.g. only recording this when it proved to be relevant for this patient, or that psychosocial and occupational problems were known to the GP under another ICPC code, such as burnout (Z29.01 Burn-out) or problem with work situation (Z05), which were not included in the present analysis.

The current study was inspired by a mass media population-based campaign that aimed to improve back beliefs of the general public and influence management of LBP by GPs in the state of Victoria in Australia [16, 17]. The Australian campaign was adapted to the Dutch context and was specifically targeted to GPs and patients with LBP. While using similar campaign messages, the results of the current study differ from the Australian study, where an improvement in GP beliefs and intended behaviour towards LBP over time was found. These differences may be due to different timings (1999 vs. 2013), and the advice received through the campaign messages might already have been widely adopted by the GPs in the current study, and therefore did not trigger changes in thinking, and thereby in behaviour. The differences in effects can also be explained by the different approaches (population-based versus targeted approach) and population (primary versus secondary prevention).

Compared to other studies using targeted interventions aimed at implementing guidelines and improving guideline adherence in LBP, the findings of the current study are in line with other research. A fairly recent cluster RCT aimed at reducing imaging referrals, showed that a theory-informed implementation strategy for a guideline did not result in actual behavioural changes among GPs [18]. A recent systematic review showed that multifaceted strategies are not more effective than usual care or minimal implementation strategies in improving professionals’ behaviour and guideline adherence [19]. The lack of success of mass-media population-based and targeted multifaceted strategies in improving GPs management of LBP in primary care, suggests that it remains difficult to change clinical practice behaviour. This is also highlighted by a Cochrane systematic review that showed varying effects of interventions aimed at improving clinicians’ imaging requests for patients with LBP [20].

Various barriers to guideline adherence have been reported previously. A systematic review on barriers to guideline adherence for LBP in primary care physicians showed that perceptions and beliefs were important barriers, and while healthcare is supposed to be evidence-oriented, the actual uptake of evidence in practice remains a challenge [21, 22]. A survey among 703 GPs in the Netherlands showed that 89% of GPs believed that following guidelines leads to improved patient care, while perceived adherence to guidelines varied between 50 and 95% [23]. Barriers to guideline adherence in this study were mostly patient-related such as their preferences [23]. Other studies found that although GPs expressed confidence in guidelines, they more often report practical barriers for adherence than other healthcare providers, and that organisational constraints (e.g. logistic problems with office hours) are the most frequently perceived environmental barrier to guideline adherence [24, 25]. A process evaluation alongside the current study showed that the GPs participating in this study experienced several barriers for guideline adherence in practice, in which contextual and organisational factors played an important role [13]. The barriers included a lack of time, lack of usable technology, and lack of trust between healthcare professionals, in line with findings in other studies [26, 27]. These and possibly other, yet unknown, barriers may have contributed to the lack of effectiveness observed in the current study. Future guideline developers, and intervention and implementation providers are recommended to take these barriers into account.

Over the past decades, ongoing debate over the effectiveness of CME on changing professional performance has been observed [28, 29]. A systematic review of 136 articles and 9 systematic reviews suggested that CME is an effective method for knowledge transition and for changing attitudes in practicing physicians, although the evidence for this conclusion was of low quality [30]. In contrast, a recent Cochrane review focusing on management of musculoskeletal conditions suggested that guideline dissemination and CME for GPs will lead to little or no improvement on guideline adherence by GPs, whereas the combination of guidelines and feedback will reduce requests for diagnostic imaging only slightly [31]. The results of the current study are in line with these conclusions, and suggest that CME might not be the most promising tool for changing clinician behaviour. However, as the current implementation strategy was multifaceted, there is no certainty as to which component was effective and to what extent. The need to develop effective ways of improving guideline adherence remains high.

### Strengths and limitations

The strength of the current study lies in the fact that the outcomes (i.e. performance indicators) were based on the review of a large number of EMRs of LBP patients. Also, the usual care group allowed for a controlled before-after analysis. One limitation is that not all GPs per general practice attended the CME training, and the use of the other components of the implementation strategy was rather low among GPs (i.e. professionals indicated that they did not use the social media platforms due to a lack of time) [13]. Due to the low attendance rates, it is uncertain whether the absence of effects is a result of theory failure or process failure. Another limitation is that the data collection method was based on routine care data, i.e. GPs were not stimulated to make more elaborate notes of LBP patient contacts for this study, and no specific guidelines exist as to what information they should note down in the records. However, this may also be a strength, because it represents actual practice and is a reliable representation of usual care. Furthermore, the performance indicators reflect registered care of all patient contacts, without a qualitative assessment of their appropriateness for a particular patient contact (e.g. whether referral was indicated according to the guideline or not), and without considering whether the contact was a first contact or a follow-up consultation (e.g. when the same patient has contacted the GP multiple times, there will be more information recorded on which indicators were scored). The performance indicators reflect the behaviour of a GP in a patient consultation to the extent to which the GP documents his or her findings and decisions in the EMR. It is not possible to fully link behaviour to clinical outcomes, which are the ultimate end points for guidelines. Bias may also have occurred due to the fact that all GPs were participating in the academic network of general practice of VUmc. They may already have been more informed about or keen to follow existing guidelines, explaining the relatively low referral rates at baseline.

### Implications for research and practice

This study does not provide evidence that the multifaceted implementation strategy in its current form should be used as a strategy for widespread implementation of guidelines in general practice in the Netherlands. Future research should investigate whether national GP behaviour has changed since 2008, reducing the need for these types of strategies. Furthermore, studies will need to invest in increasing GP engagement and attendance at training, while also targeting more high-risk practices, i.e. practices where the number of referrals for LBP is high and there is room for improvement.

## Conclusion

A multifaceted implementation strategy aiming to improve guideline adherence among GPs in managing patients with LBP, did not result in relevant reductions of referral rates to medical specialist care or diagnostic imaging as measured by performance indicators. However, baseline referral rates were already low, possibly leaving little room for improvement. Inquiries for psychosocial and occupational risk factors remained low leaving room for improvement.

## Abbreviations

ANH-VUmc: Academic network of general practice of VU University medical centre
CI: Confidence interval
CME: Continuing medical education
CT-scan: Computed tomography scan
EMR: Electronic medical record
GEE: Generalized estimating eqs.
GP: General practitioner
ICPC: International classification of primary care
LBP: Low back pain
MRI: Magnetic resonance imaging
NTR: Netherlands trial register
OR: Odds ratio
RCT: Randomised controlled trial
SD: Standard deviation
WMO: Medical research involving human subjects act (Netherlands)
X-ray: Rontgen radiation
